# The Combined Effects of Cold Foot Bath and Lavender Oil Inhalation on Autonomic Variables in Healthy Volunteers: A Randomized Controlled Trial

**DOI:** 10.7759/cureus.77055

**Published:** 2025-01-07

**Authors:** Kavya C Gowda, Sujatha KJ, Prashanth Shetty

**Affiliations:** 1 Natural Therapeutics, Sri Dharmasthala Manjunatheshwara (SDM) College of Naturopathy and Yogic Sciences, Ujire, IND; 2 Nutrition and Dietetics, Sri Dharmasthala Manjunatheshwara (SDM) College of Naturopathy and Yogic Sciences, Ujire, IND

**Keywords:** aromatherapy, autonomic function, cold foot bath, hydrotherapy, lavender oil

## Abstract

Background and aim: An essential function of the autonomic nerve system is to regulate physiological processes and stress responses in the body. Cold foot baths and aromatherapy with lavender oil each influence autonomic functions, but their combined effect in healthy individuals is unknown. The purpose of this study is to look into how autonomic variability in healthy volunteers is affected by both inhaling lavender oil and taking cold foot baths.

Methods: A total of 60 healthy individuals were randomized to be placed in either the control group (n=30) or the experimental group (n=30) and were instructed to attend a single designated session. The control group underwent a 20-minute cold foot bath, while the experimental group received a 20-minute cold foot bath combined with lavender oil inhalation. Assessments were carried out before, during, and after the interventions.

Outcome measures: To evaluate autonomic variables, we monitored galvanic skin response (GSR) and Heart Rate Variability (HRV) using an equivital belt. Furthermore, we measured blood pressure (BP) and mean arterial pressure (MAP) before, during (20 minutes) the intervention, and after a 10-minute resting period; post-intervention measurements were taken.

Results: Repeated measures analysis revealed a significant difference in standard deviation of normal-to-normal intervals (SDNN), pNN50, and heart rate (HR) for time-domain variables (p<0.05), whereas the frequency-domain analysis showed a significant difference over time in LF/HF, LF, and HF (p<0.05). When these were compared between the groups, a significant difference was observed only in LF and HF (p<0.04). Additionally, a statistically significant difference (p<0.001) in diastolic and systolic blood pressure between the groups was noted.

Conclusion: The combination of a cold foot bath and lavender oil inhalation may modulate autonomic activity, promoting relaxation by vagal balance in healthy individuals.

## Introduction

Heart rate variability (HRV), which is a measure of neurocardiac function, is produced by interactions between the brain and heart along with dynamic non-linear autonomic nervous system (ANS) processes [1]. Numerous physiological processes, including blood pressure, respiration regulation, and heart rate, are crucially regulated by the autonomic nervous system [2]. Blood pressure (BP), gas exchange, heart rate, autonomic balance, and vascular tone - the width of blood vessels that control BP - are a mere few of the physiological processes that are reflected in HRV [3].

Hydrotherapy is a natural healing practice that has been used in antiquity civilizations like India, China, Egypt, and other regions. It involves using water in various forms, such as ice and steam, internally and externally to treat or enhance health in different conditions, using different temperatures, pressures, durations, and locations [4]. This therapy has the following three primary health impacts: thermal, mechanical, and chemical. Thermal effects can be achieved through different temperature ranges such as heat therapy (35°-40°C), body temperature regulation (32°-34°C), or cold therapy (8°-10°C) [5]. These temperature variations can have individual or combined effects on the body. Cold therapy is frequently linked to vasoconstriction and pain alleviation, whereas the benefits of heat therapy are typically associated with vasodilation and increased blood flow [6]. Aromatherapy is a treatment used in complementary and alternative medicine that utilizes essential oils, primarily derived from volatile liquid plant components and other plant-derived aromatic compounds [7-9,10]. Among the most often used essential oils in aromatherapy, lavender oil is used to treat a variety of medical ailments, such as anxiety, depression, insomnia, spasms, pain, and headaches [10-12]. Lavender oil is originally from the Mediterranean region and is a member of the mint family Lamiaceae, specifically the genus Lavandula (*Lavandula angustifolia* Mill). The lavender essential oil typically contains "linalyl acetate, β-linalool, and β-caryophyllene" [13]. Essential oils are used in different ways such as topical skin application, bathing, massage, inhalation, and compression [8]. Inhalation is a key method in aromatherapy as aromas can impact physiology, behavior, and emotion [14]. According to a prior study, breathing in lavender oil lowered skin temperature, heart rate, and blood pressure - all signs of lower autonomic arousal. Participants in the lavender oil group also mentioned feeling more alert, invigorated, and at ease [15]. Essential oils can be inhaled to alter neurotransmission pathways that impact emotions and activate the brain's production of mood-regulating neurotransmitters like dopamine and serotonin [16,17]. To our knowledge, there is no evidence available testing the combined effect of cold foot baths and lavender oil inhalation on autonomic variability in healthy volunteers.

This study aimed to assess the combined effect of cold foot baths and lavender oil inhalation on autonomic functions.

## Materials and methods

Study design and setting

**Figure 1:**
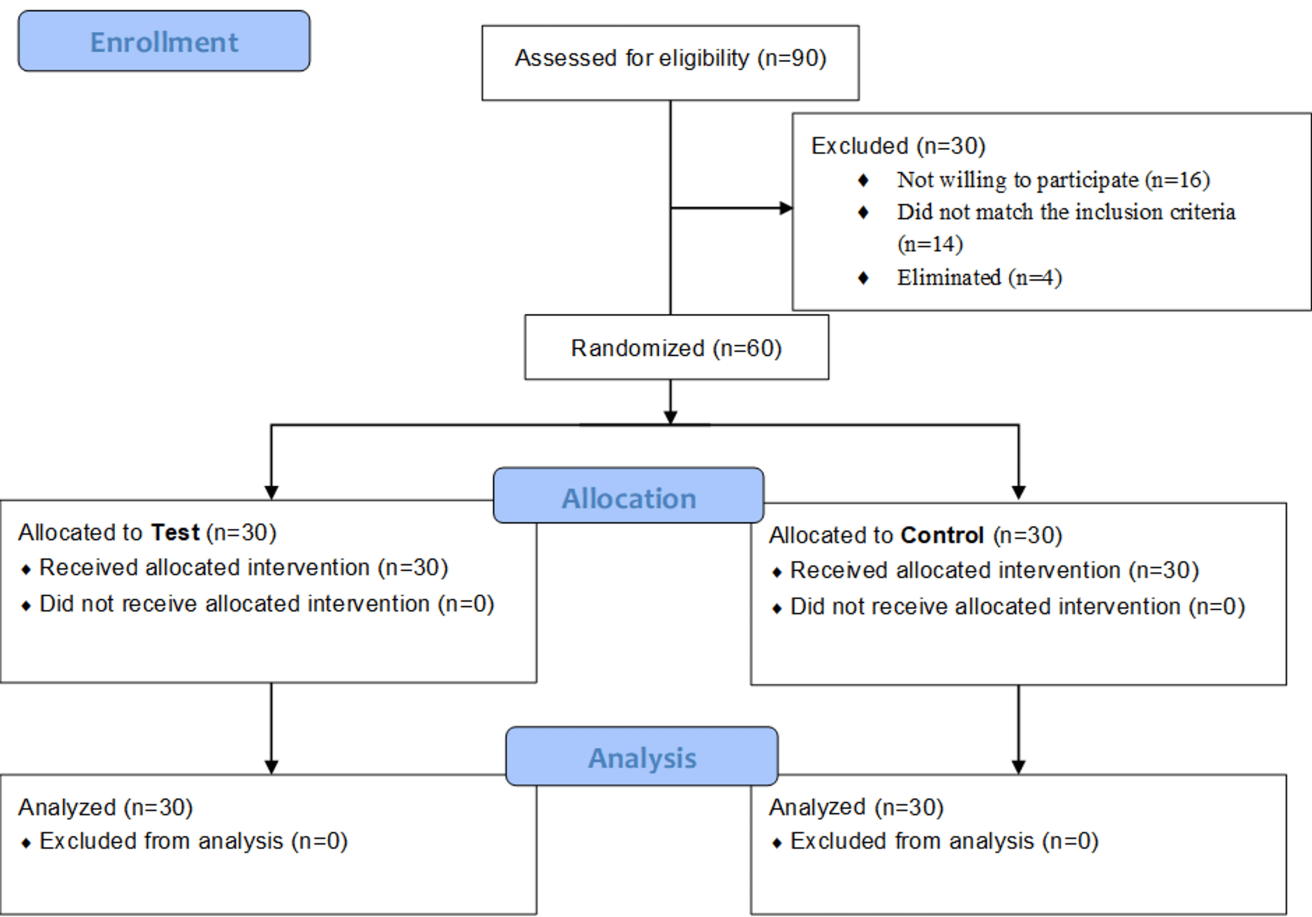
CONSORT flow chart of the study. CONSORT: Consolidated Standards of Reporting Trials

The study is an open-label, single-center, randomized controlled trial, designed according to Consolidated Standards of Reporting Trials (CONSORT) guidelines as shown in Figure 1. Subjects were recruited from a holistic health center in the southern part of India. The recruitment period was from January 2024 to June 2024.

Participants

The inclusion criteria were as follows: males and females aged 18-28 years, and subjects who have signed the informed consent form and are willing to participate in the study. The subjects were excluded if they were weak and debilitating individuals, had any systemic illness, subjects with open wounds on their legs, and consumed caffeine two hours before the intervention. The subjects who were recruited were randomly divided into two groups as follows: the experimental group, which received a cold foot bath with lavender oil inhalation for 20 minutes, and the control group, which received only a cold foot bath. Assessments were conducted before, during, and after the intervention.

Sample size calculation

The sample size was determined using G*Power 3.1.9 (Düsseldorf, Germany: Heinrich Heine University Düsseldorf) for 80% power, a two-sided p-value, a 1:1 allotment ratio, a 5% significance level, and an effect size of 0.8. The total sample size was determined to be 52; accounting for a 10% attrition rate, the sample size increased to 60 (30 subjects per group).

Randomization and blinding

A computerized randomization process was employed to ensure an unbiased assignment of participants to either the experimental group or the control group. This method aimed to minimize selection bias and maintain the integrity of the study's design. Additionally, the data analyst responsible for processing and analyzing the results was blinded to the group allocation of the participants. This blinding procedure was implemented to reduce the risk of analytical bias and to enhance the reliability and validity of the study outcomes.

Intervention

Group 1

Participants were instructed to submerge their lower limbs (both left and right) for 20 minutes in a specially prepared tub of cold water (18°-15°C), covering their toes, ankle joints, feet, and portions of their legs (i.e., up to the origin of the Achilles tendon's). The individuals' water levels vary slightly from one another because the water was selected according to the following standards: water that was adequate to completely submerge the targeted lower limb regions (up to the origin of the Achilles tendon). The water temperature was measured using a water thermometer, and lavender oil inhalation was administered using an aroma diffuser for 20 minutes.

Group 2

Participants were instructed to submerge their lower limbs (both left and right) for 20 minutes in a specially prepared tub of cold water (18°-15°C), covering their toes, ankle joints, feet, and portions of their legs (i.e., up to the origin of the Achilles tendon). The individuals' water levels vary slightly from one another because the water was selected according to the following standards: water that was adequate to completely submerge the targeted lower limb regions (up to the origin of the Achilles tendon). The water's temperature was measured by using a water thermometer.

Assessment

The subjects were told to wear a life monitor model EQ02+ made by Equivital (Cambridge, UK) [18]. The Equivital LifeMonitor is a wearable gadget that resembles a vest and has an electrical sensor module cradled on its side. Participants were instructed to charge the LifeMonitor at least every other night and to remove it during any activity involving water. Wear time was calculated as the average amount of time the LifeMonitor was worn in a single day, based on the number of days it was worn. The Equivital LifeMonitor recorded galvanic skin response (GSR), respiratory rate (RR), HR, and heart rate variability (HRV) [18]. The ambiguous life monitor was used to measure HR, HRV, GSR, and RR before, during, and after the intervention. In order to prevent error, blood pressure and mean arterial pressure (MAP) readings were manually obtained using a sphygmomanometer while the participant was comfortably seated [19]. Prior to their assessments, all individuals were instructed to abstain from alcohol, cigarettes, coffee/tea, food, and exercise for at least half an hour [20]. Additionally, they were told to keep breathing normally and to avoid limb and other bodily motions to steer clear of the artifacts in motion [21,22].

Statistical analysis

The Jamovi project (2022) was utilized. Jamovi (v2.3) is a statistical analysis program. Statistics were defined as significant at p<0.05. The Shapiro-Wilk test was used to determine whether the data were normal. The data were presented as mean±standard deviation. RMANOVAs or repeated measurement ANOVAs were used for the analyses. The equality of the variances of the differences between levels of the repeated measurements component was investigated using Mauchly's test of sphericity. The degrees of freedom in the F-tests were corrected using the Greenhouse-Geisser approach because the result showed that the assumption of sphericity was broken, p<0.001. The significance level for each analysis was established at α=0.05. The Mann-Whitney U test was used to evaluate the post values between groups.

## Results

For eligibility, 90 healthy volunteers were screened. Sixteen volunteers opted out, and 14 did not meet the inclusion criteria. Four potential candidates were excluded before randomization. At first, a total of 60 individuals were registered and allocated at random; no participants were abandoned or lost in the follow-up. There were 60 participants in the final sample (group 1: n=30, group 2: n=30). Both groups are made up of both males and females, and both have an average age of 24 years. Group 1's average BMI is 23.8±1.47 kg/m^2^, whereas group 2's average BMI is 22.68±1.25 kg/m^2^. No significant difference was noted at the baseline of HRV and GSR.

Galvanic skin response was as follows: there was no significant difference between sessions, states, or groups in their interaction for GSR (p>0.05). Time domain analysis of heart rate variability was as follows: when the time domain variables of HRV were compared during the 20 minutes of intervention, a significant difference was observed (p<0.05) in SDNN, pNN50, RMSSD, and average heart rate over time. However, no significant difference was found between groups during the 20-minute intervention. Additionally, when the post-intervention values were compared between groups, no significant difference was observed, as shown in Table 1.

**Table 1:** Comparing the outcomes between group 1 and group 2. P<0.05 is considered statistically significant. All values are presented in mean±standard deviation. RM-ANOVA: Repeated Measure Analysis of Variance; SBP: systolic blood pressure; DBP: diastolic blood pressure; MAP: mean arterial pressure; GSR: galvanic skin response; HRV: heart rate variability; Average RR: average of R-R interval; SDRR: standard deviation of R-R interval; HR: heart rate; RMSSD: The square root of the mean of the sum of the squares of differences between adjacent RR intervals; pRR50: proportion derived by dividing RR50 by the total number of RR intervals; LF: low-frequency band of HRV; HF: high-frequency band of HRV; LF/HF: ratio of low frequency to high frequency; SD: standard deviation

Outcomes	Between group				RM-ANOVA	
	Group 1 (n=30)	Group 2 (n=30)	z/t	p-value	Time F (p-value)	Group F (p-value)
Hemodynamic						
SBP (mmHg)	106.43±9.26	118.8±11.76	t=4.52	<0.001	-	-
DBP (mmHg)	69.46±7	80.2±9.16	z=4.26	<0.001	-	-
MAP (mmHg)	81.76±7.2	92.83±9.18	t=5.19	<0.001	-	-
GSR	2.77±2.44	1.73±1.3	z=2.62	0.009	-	-
HRV						
Average RR (ms)	784.89±69.15	759.5±171.42	z=0.01	0.99	1.64 (0.16)	0.7 (0.4)
SDNN (ms)	58.11±19.23	55.46±19.45	z=0.01	0.99	12.96 (<0.001)	0.02 (0.86)
HR (b/min)	77.49±7.03	77.2±10.7	t=0.12	0.9	15.57 (<0.001)	0.31 (0.57)
RMSSD (ms)	46.12±20.68	41.49±20.85	t=0.86	0.39	8.5 (<0.001)	0.17 (0.67)
pRR50 (%units)	25.16±17.38	22.43±17.65	z=0.65	0.52	7.4 (<0.001)	0.005 (0.94)
LF (nu)	50.3±17.62	59.82±18.48	z=1.95	0.04	3.37 (0.01)	2.37 (0.12)
HF (nu)	49.04±16.7	39.82±18.11	z=1.9	0.04	3.06 (0.01)	2.11 (0.15)
LF/HF	1.35±1.05	2.54±3.27	t=2.02	0.05	1.77 (0.13)	2.6 (0.11)

Frequency domain analysis was as follows: the frequency domain variables of HRV during the 20 minutes of the intervention showed a significant difference (p<0.05) over time only in LF. When the post-intervention values were compared between groups, a significant difference was observed in LF and HF (Table 1).

The between-group comparison showed significant differences in SBP, DBP, MAP, and GSR when the post-intervention values of the groups were compared. However, only the LF and HF frequency domains in HRV showed a significant difference (both p<0.05) post-intervention (Table 1).

## Discussion

The combined effects of inhaling lavender oil and taking a cold foot bath on autonomic variability in healthy participants were examined in this study. Particularly in the LF and HF frequency domains, the results revealed notable alterations in group 1 hemodynamic responses and HRV. However, there were no significant differences observed in HR, SDRR, and RMSD of HRV. This suggests that the combination of cold foot baths and lavender oil inhalation may not have a significant impact on these specific HRV variables in healthy volunteers.

The equilibrium between the parasympathetic and sympathetic nervous systems is reflected in HRV and their impact on heart function [21,22]. In HRV, low-frequency power (LF) exhibits both sympathetic and parasympathetic effects, while high-frequency power (HF) indicates parasympathetic activity. One frequent indicator of sympatho-parasympathetic balance or sympathetic modulation is the ratio of LF to HF (LF/HF) [23,24]. Our study demonstrates an increase in the LF/HF ratio, indicating sympatho-parasympathetic balance. GSR or galvanic skin response is an electrodermal measure of sympathetic nerve activity.

Vasodilation in the deeper vascular system caused by cold exposure to a tiny surface area increases blood flow to the tissues underneath the exposed area. This vascular response's primary goal is to keep the deep tissue's temperature constant [23]. The cold pressor test is a common sympathetic activation test used to evaluate cardiovascular responsiveness by measuring the blood pressure response to an external cold stimulus [24]. Applying cold, typically cold water, to the tissue can serve as a simple, non-invasive autonomic challenge that triggers the sympathetic discharge, mediated centrally through activation of cutaneous thermoreceptors. Peripheral vasoconstriction is induced by the increased sympathetic outflow, which raises pulse pressure and cardiac output [25,26]. The cold pressor test highlights the importance of the central nervous system in blood pressure control and has been used to identify autonomic traits in both healthy and sick persons that result in higher heart rate and blood pressure [27]. As a result, the skin temperature of the fingers and toes declines quickly and dramatically to a level that is similar to the ambient temperature due to vasoconstriction and the high surface area-to-volume ratio [28].

The essential lavender oil contains "linalyl acetate, β-linalool, and β-caryophyllene" [13]. According to the pharmacokinetic properties of linalool, Yamada et al. demonstrated that the "lipophilic nature of linalool" is appropriate to carry the substance over the blood-brain barrier [29]. Upon accomplishing the brain, linalool can bind to the "gamma-aminobutyric acid (GABA) receptors," many likely "benzodiazepines," resulting in relaxing and sedative effects. In a study, it was found that linalool could enhance the effects of GABA, the primary inhibitory neurotransmitter in the human brain, particularly in the amygdala, a subcortical brain region involved in emotional responses to the environment [30]. The impact of linalool on the amygdala may elucidate the mood-altering effects of lavender. Previous research suggests that lavender oil can have a positive effect on parasympathetic neural activity when used in a foot bath [31]. Inhaling lavender oil significantly increases theta "(4-8 Hz) and alpha (8-13 Hz)" activity in the brain. Diego et al. found that inhaling lavender oil greatly enhanced frontal alpha power [32]. A previous study showed that inhaling lavender oil resulted in a relaxation effect and increased alpha-wave activity in the brain [15].

Overall, these results indicate that the use of lavender oil inhalation with cold foot baths as an intervention may have a synergistic effect on autonomic variability in healthy individuals. Further research is needed to explore the potential mechanisms underlying these effects and to determine the clinical implications for using this combination therapy in the management of stress, anxiety-related disorders, and cortisol levels. No adverse effects like discomfort, cold intolerance, or allergic reactions to lavender oil were noted in the study. So, this combined intervention can be considered a non-invasive therapeutic tool to modulate autonomic functions.

It must be acknowledged that this study had limitations. This study was conducted on healthy volunteers only. This makes it difficult to extrapolate the findings to individuals with specific health conditions or disorders, as their autonomic variability may differ. Future research should include participants with different health backgrounds to determine the generalizability of the results. Another limitation is the small sample size of this study. A bigger sample size would boost the study's statistical power and enhance the results' generalizability. Furthermore, a longer duration of intervention and follow-up period would allow for a more comprehensive assessment of the effects of the combined intervention on autonomic variability, and a true placebo is needed to validate the results. The combined intervention's long-term effects on autonomic variability were not evaluated in this study. Future research should include a longitudinal follow-up to determine if the effects are sustained over time or if they diminish after repeated use.

## Conclusions

In conclusion, this study supports the use of cold foot baths and lavender oil inhalation as effective, non-invasive methods for fostering relaxation and alleviating stress by positively influencing the autonomic nervous system. Specifically, these practices are shown to enhance parasympathetic activity, which is associated with the body's "rest-and-digest" functions, and improve autonomic variability, an indicator of the nervous system's adaptability and overall health. Incorporating such practices into daily routines may provide individuals with an accessible and natural approach to managing stress, promoting emotional well-being, and supporting physical health by restoring balance to the autonomic nervous system. These findings highlight the potential of simple, holistic interventions to address modern lifestyle challenges and improve quality of life.
